# Supplementary material for: Unpleasant but effective: Newspaper coverage of cancer screening and cancer in the Netherlands from 2010 to 2022
Source: PLoS One. 2025 Oct 22;20(10):e0334121. doi: 10.1371/journal.pone.0334121 (PMC12543187; doi:10.1371/journal.pone.0334121)
Supplement: S1 File — (DOCX) [file pone.0334121.s001.docx]

# Supporting information 1: News media outlets and search string

## Newspaper outlets

*National*

AD/Algemeen Dagblad, AD/Algemeen Dagblad.nl, De Groene Amsterdammer, De Telegraaf, De Telegraaf.nl, de Volkskrant, De Volkskrant.nl, Het Parool, Het Parool.nl, Metro NL, Metronieuws.nl, Nederlands Dagblad, NRC, NRC Handelsblad, NRC.nl, Reformatorisch Dagblad, Spits, Trouw, Trouw.nl

*Regional*

BN De Stem.nl, BN/DeStem, Brabants Dagblad, Brabants Dagblad.nl, Dagblad De Limburger, Dagblad De Limburger.nl, Dagblad de Limburger, Dagblad van het Noorden, Dagblad van het Noorden.nl, De Gelderlander, De Gelderlander.nl, De Noordoostpolder, De Stentor, De Stentor.nl, De Twentsche Courant Tubantia, Eindhovens Dagblad, Eindhovens Dagblad.nl, Leidsch Dagblad, Leidsch Dagblad.nl, Limburgs Dagblad, Noordhollands Dagblad, Noordhollands Dagblad.nl, Provinciale Zeeuwse Courant, PZC.nl, Tubantia.nl

*Local*

AD/Amersfoortse Courant, AD/De Dordtenaar, AD/Groene Hart, AD/Haagsche Courant, AD/Rivierenland, AD/Rotterdams Dagblad, AD/Sportwereld Pro, AD/Utrechts Nieuwsblad, Almere Vandaag, Alphen.cc, Asser Courant, Balkster Courant, De Feanster, Bolswards Nieuwsblad, Coevorder Courant, De Betuwe, De Brug Nijmegen, De Gooi en Eemlander, De Gooi en Eemlander.nl, De Maasroute, Deventer Post, Drachtster Courant, Eemsbode, Emmer Courant, Extra Nieuws Leerdam, Flevopost Dronten, Flevopost Lelystad, Franeker Courant, Friesch Dagblad, Goudsche Courant, Groninger Gezinsbode, Groot Rijswijk, Haarlems Dagblad, Haarlems Dagblad.nl, Heerenveense Courant, Hoogeveensche Courant, HSkrant, Huisaanhuis, Huisaanhuiskranten de Persgroep, IJmuider Courant, IJmuider Courant.nl, IJssel Lekstreek Capelle, Jouster Courant ZuidFriesland, Kanaalstreek Ter Apelerer, Courant, Krant van Midden Drenthe, Leeuwarder Courant, Leeuwarder Courant.nl, Meppeler Courant, Nieuwe Ooststellingwerver, Nieuwsblad NoordoostFriesland, Noorderkrant, Oost Gelre, Papendrechts Nieuwsblad, Roder Journaal, Sneeker Nieuwsblad, Stadsnieuws, Staphorster, Steenwijker Courant, Stem van Dordt, Valkenswaards Weekblad, Veendammer, Twents Volksblad, Westervelder Wolder Courant, Bennekoms Nieuwsblad, Stadsblad, Apeldoorns Stadsblad, Zenderstreeknieuws Montfoort Oudewater, Goudse Post, Streekblad, Groot Eindhoven, Ermelos weekblad, Groot Roosendaal, Amersfoort Nu, Hengelos Weekblad, Westerkwartier, Huis aan Huis, Stellingerwerf, Almelos Weekblad, Alphens Nieuwsblad, Amersfoort NU, Arnhemse Koerier, Barneveld Vandaag, Bodegraafs Nieuwsblad, Botlek Hoogvliet, Botlek Hoogvliet en Albrandswaard, Botlek Spijkenisse, De Bevelander, De Botlek 1, De Brug, De Faam, De Kempenaer, De Kombinatie Hendrik Ido Ambacht, De Kombinatie Zwijndrecht, De Nieuwe Krant, De Parel van Brabant, De Posthoorn, Segbroek, De Rijnpost, De Schakel, De Toren, De Vonk, De Waalkanter, De Waalkanter ed DrutenWijchen, de Weekkrant NoordoostTwente, De Weekkrant Papendrechts Nieuws, Delftse post, Doetinchems Vizier, Duiven Post, ed Gorinchem, Edese Post, Gouwe Koerier, Groot Bergen op Zoom, Groot Vlaardingen, Haaksberger Koerier, Het Zuiden barendrecht, Het Zuiden Ridderkerk, Huis aan Huis Elburg, Huis aan Huis Enschede, IJssel Lekstreek Krimpen, IJssel en Lekstreek Capelle, IJssel en Lekstreek Krimpen, IJsselstreek Vizier, Koerier Beuningen, Nieuwe Dinkellander, Nunspeet Huis aan Huis, Oirschots Weekjournaal, Oude IJsselstreek Vizier, Parel van Brabant, Stad Wageningen, Stellingwerf, Traverse, Vechtstroom, Veldhovens Weekblad, Veluws Nieuws, Veluws Nieuws editie Epe, Westerkwartier, Westervoort Post, Zevenaar Post

## Search string Lexis Nexis

(baarmoederhalskanker* OR “baarmoederhals kanker*”OR cervixcarcinoom OR hpv OR papillomavi* OR cervix OR cervic* OR borstkanker* OR “borst kanker*” OR mammacarcinoom OR (knobbel* AND borst*) OR borsttumor* OR darmkanker OR “darm kanker” OR “colorectoraal carcinoom” OR crc OR coloncarcinoom OR dikkedarmkanker OR “colorectale kanker” OR prostaatkanker* OR “prostaat kanker*” OR prostaatcarcinoom OR PSA* OR “prostaat specifiek antigeen” OR longkanker* OR “long kanker*” OR longcarcinoom OR *kanker* OR *tumor* OR *carcino*)

AND

(uitstrijk* OR colposcopie* OR swab* OR zelfafnameset* OR zelfafnametest* OR zelftest* OR thuistest* OR mammografie* OR borstfoto* OR mammogram OR (röntgenfoto* AND borst*) OR borstonderzoek* OR ontlastingstest* OR poeptest* OR colonoscopie* OR coloscopie* OR PSA-test OR “PSA test” OR screen* OR kijkonderzoek* OR bevolkingsonderzoek* OR screeningsorganisatie* OR ((longkanker* OR “long kanker*” OR longcarcinoom) AND (“vroeg* opspor*” OR “vroeg* ontdek*” OR “vroeg* diagnos*”)))
