# Supplementary material for: Unpleasant but effective: Newspaper coverage of cancer screening and cancer in the Netherlands from 2010 to 2022
Source: PLoS One. 2025 Oct 22;20(10):e0334121. doi: 10.1371/journal.pone.0334121 (PMC12543187; doi:10.1371/journal.pone.0334121)
Supplement: S2 File — (DOCX) [file pone.0334121.s002.docx]

# Supporting information 2: Rouge test scores for duplicate detection

**S2 Fig 1. F1-scores for a test set of 100 randomly selected articles from the corpus with Rouge unigram, bigram, and longest common subsequence (LCS) scores at different thresholds.**


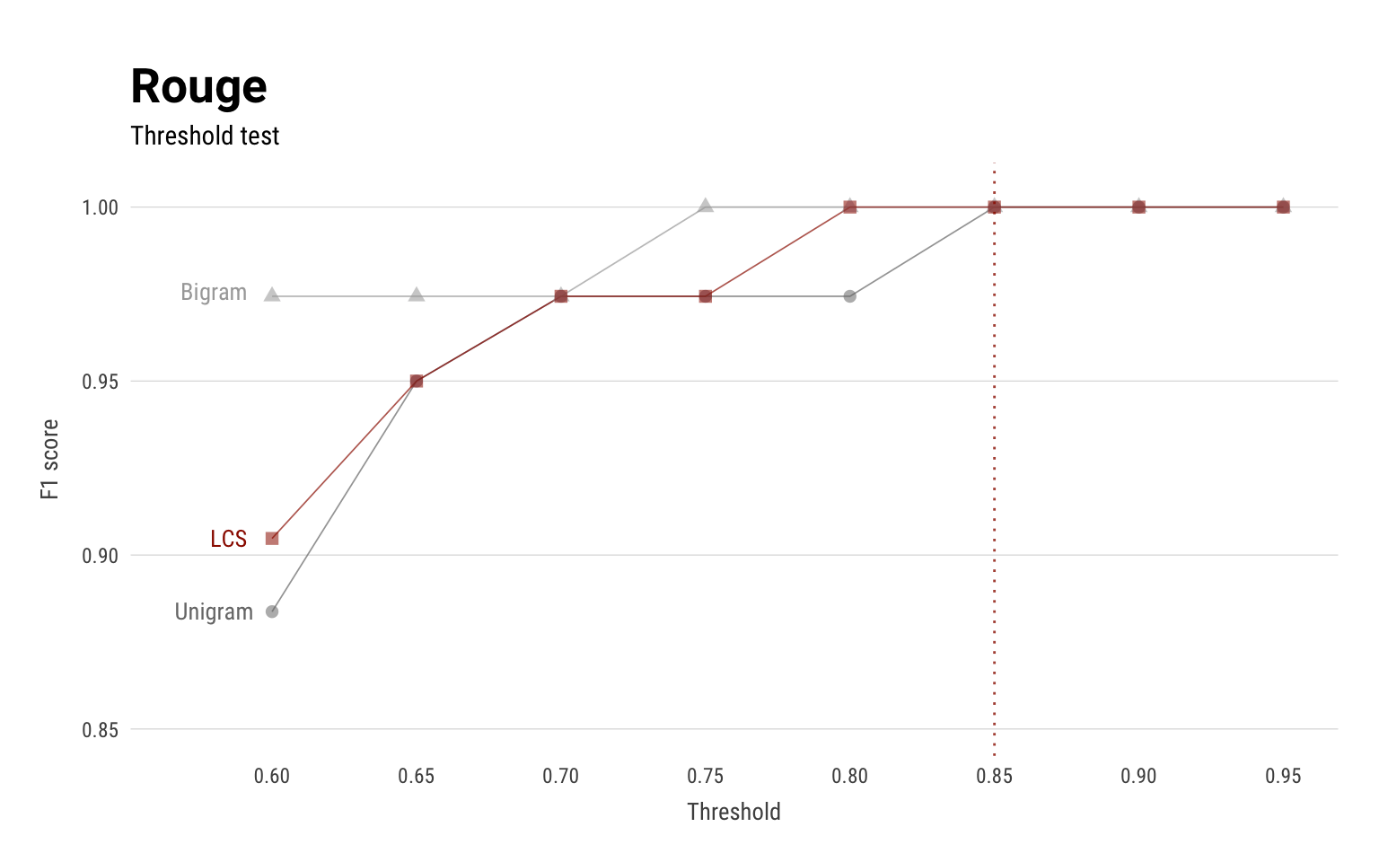


** Please note that the y-axis is truncated for readability purposes.*

*NB:* We aimed to find a threshold that was as low as possible and gave a reliable output. We purposely did not use the highest possible thresholds to prevent incorrectly assigning single articles as duplicates in a larger sample (i.e., false positives). LCS was used for duplicate detection as unigram scores appeared unreliable for shorter news articles, and the metric is similar to the task at hand, i.e., finding text with sentences that overlap to a very large extent. Although the LCS was already reliable at a threshold of .8, we eventually chose a threshold of .85 since the .8 threshold appeared to include some single articles as duplicates when running the final code.
