# Supplementary material for: Unpleasant but effective: Newspaper coverage of cancer screening and cancer in the Netherlands from 2010 to 2022
Source: PLoS One. 2025 Oct 22;20(10):e0334121. doi: 10.1371/journal.pone.0334121 (PMC12543187; doi:10.1371/journal.pone.0334121)
Supplement: S3 File — (DOCX) [file pone.0334121.s003.docx]

# Supporting information 3: Frequency distribution of the news framing for all sources

**S3 Table 1. Frequencies of news frames when a source is cited or referred to in an article.**

|  | **Health care provider**  (*n* = 175) | **Science**  (*n* = 160) | **Health organization**  (*n* = 159) | **Politics**  (*n* = 69) | **Personal**  (*n* = 142) | **Other**  (*n* = 61) |
| --- | --- | --- | --- | --- | --- | --- |
| *Type of framing* | *n* (%) | *n* (%) | *n* (%) | *n* (%) | *n* (%) | *n* (%) |
| **Screening effectiveness** |  |  |  |  |  |  |
| Explicitly effective | 53 (30.3) | 42 (26.3) | 52 (32.7) | 18 (26.1) | 45 (31.7) | 8 (13.1) |
| Implicitly effective | 48 (27.4) | 34 (32.3) | 58 (36.5) | 26 (37.7) | 56 (39.4) | 23 (37.7) |
| Disputed | 55 (31.4) | 66 (41.3) | 31 (19.5) | 18 (26.1) | 24 (16.9) | 14 (23.0) |
| Neutral | 19 (10.9) | 18 (11.3) | 18 (11.3) | 13 (18.8) | 17 (12.0) | 16 (26.2) |
| **Cancer risk** |  |  |  |  |  |  |
| High | 41 (23.4) | 32 (20.0) | 44 (27.7) | 13 (18.8) | 39 (27.5) | 10 (16.4) |
| Low | 10 (5.7) | 9 (5.6) | 5 (3.1) | 0 (0.0) | 8 (5.6) | 10 (16.4) |
| Neutral | 124 (70.9) | 119 (74.4) | 110 (69.2) | 56 (81.2) | 95 (66.9) | 1 (1.6) |
| **Cancer consequences** |  |  |  |  |  |  |
| Severe | 99 (56.6) | 80 (50.0) | 90 (56.6) | 23 (33.3) | 109 (76.8) | 25 (41.0) |
| Not severe | 5 (2.9) | 3 (1.9) | 3 (1.9) | 3 (4.4) | 10 (7.0) | 5 (8.2) |
| Neutral | 71 (40.6) | 77 (48.1) | 66 (41.5) | 43 (62.3) | 23 (16.2) | 31 (50.8) |
| *NB:* for each source, it was indicated whether they were *present* or *absent* in a news article. For each source, percentages represent whether a framing subcategory is present within all news articles that refer to the source category. For instance, for all news articles that refer to health care providers (n = 175), 55 screening effectiveness frames are categorized ‘explicitly effective’ (31.4%). | | | | | | |
