# Supplementary material for: Unpleasant but effective: Newspaper coverage of cancer screening and cancer in the Netherlands from 2010 to 2022
Source: PLoS One. 2025 Oct 22;20(10):e0334121. doi: 10.1371/journal.pone.0334121 (PMC12543187; doi:10.1371/journal.pone.0334121)
Supplement: S4 File — (DOCX) [file pone.0334121.s004.docx]

# Supporting information 4: News events related to cancer screening

**S4 Table 1. News events related to cancer screening**

|  | **Date** | **Explanation** |
| --- | --- | --- |
| **Breast cancer** |  |  |
| Less cancer diagnoses | December 2020 – February 2021 | During the COVID-19 pandemic, the cancer screening program is temporarily put on hold. In December, health care providers alarm that screening should be restarted, and in February, the Dutch cancer registration announces that the number of (breast) cancer diagnoses has decreased since the start of the pandemic. |
| Breast cancer month | October each year | Some smaller peaks in the volume of breast cancer screening news can be detected in October of each year during the international Breast Cancer Awareness Month. |
| **Cervical cancer** |  |  |
| HPV vaccination campaign | March 2010 | A Dutch study (Gök et al., 2010) shows that the introduction of a home kit for cervical cancer screening can increase coverage. |
| Conflict of interest case | June 2015 | A clinician involved in the advice for the introduction of a new screening method in the cervical cancer program is involved in a possible conflict of interest case for concealing information about receiving financial support from industry. |
| Advice home kit | October 2021 | The health council releases an advice to send a home kit (rather than a home kit application form) to women eligible for cervical cancer screening. |
| **Colon cancer** |  |  |
| Screening announcement | May 2011 | The minister of health announces the introduction of a new colon cancer screening program for all Dutch citizens between 55 and 75 years of age. |
| Launch screening program | January 2014 | The colon cancer screening program is officially launched. |
| **Lung cancer** |  |  |
| Publication of RCT results | January 2014 | The results of a study financed by the US Preventive Services task force in which Dutch researchers were involved (de Koning et al., 2014) shows that the mortality rate of lung cancer can decrease when older and former smokers receive an annual CT-scan. |
| Test lung cancer screening | August 2022 | A European study, led by researchers from a Dutch hospital, is announced to start to test the effectiveness of lung cancer screening, for which 400.000 people are invited to participate. |
