# Supplementary material for: Unpleasant but effective: Newspaper coverage of cancer screening and cancer in the Netherlands from 2010 to 2022
Source: PLoS One. 2025 Oct 22;20(10):e0334121. doi: 10.1371/journal.pone.0334121 (PMC12543187; doi:10.1371/journal.pone.0334121)
Supplement: Attachment — Submitted filename: Response to Reviewers.docx [file pone.0334121.s006.docx]

Dear editors, dear reviewers,

We would like to thank you for the opportunity to revise and resubmit our work and for the valuable suggestions you provided, which were very helpful in improving the manuscript. We carefully considered the reviewer comments, and we are confident that the revised version of the manuscript resolved the raised concerns and presented our reasoning more appropriately.

In the revised paper, we discuss newspaper consumption in the Netherlands in the beginning of our Theory section to help readers contextualize the study better. Based on suggestions made, we reflect more on the impact of media, and the role that social media can have next to news media, in the discussion.

Issues with the figures are resolved and all images are now of better quality, and we have changed the font to Arial, which is described as one of the suitable fonts on the journals’ website.

The OSF links in the previous manuscript were anonymized view-only links. However, since the reviews were not (double) blinded, we now changed these links to the final, non-anonymized ones.

Please find a detailed response to all suggestions and a description of all major changes made within the manuscript below.

Kind regards,

Authors

**Editor**

1. Please ensure that your manuscript meets PLOS ONE's style requirements, including those for file naming.

*Thank you for this remark, we altered the file names for our documents accordingly.*

2. Please include your amended Funding Statement within your cover letter. We will change the online submission form on your behalf. Please include your amended statements within your cover letter; we will change the online submission form on your behalf.

*Thank you for these remarks, please find our revised funding statement below. We removed the statement from our manuscript file(s) and updated the one below to align with the information that should be in there accordingly.*

Disclosure statement: This research is part of the SENTENCES (Social mEdia aNalysis To promotE cancer Screening) project and is funded by ZonMw under grant project number 555004205. Project leader for this project is prof. dr. HHJ Das, who is also an author of this paper. <https://projecten.zonmw.nl/nl/project/sentences-social-media-analysis-promote-cancer-screening>. There was no additional external funding received for this study.

3. Data availability

*Thank you for sharing your concerns. We do not know why this miscommunication occurred, but the data is available on OSF via the link in the data availability statement in the manuscript.*

4. Please include captions for your Supporting Information files at the end of your manuscript, and update any in-text citations to match accordingly.

*Thank you for this comment. We added the list of captions of the supporting information files at the end of the manuscript and altered the references in the text-body of the manuscript.*

**Reviewer #1**

1. Table:1, The author should make the table with suitable references.

*Thank you for this remark. We now refer to the table in the running text of the manuscript.*

*Line 269*

Table 1 lists the news framing categories with examples from the corpus.

2. Fig.1, 2, 3 and 4, the authors should keep legible fonts and clear TIFF images for all the figures.

*Thank you for this remark. We altered the font in the images to Arial, exported our files as high-quality .svg files and used the tool that the journal provides to convert our files to .tiff files while maintaining image quality as much as possible.*

3. There are several typos throughout the manuscript; the authors should rectify accordingly.

*Thank you for pointing this out. For the revised version we went through the manuscript and rectified the typos.*

4. Font sizes were not even throughout the manuscript; also, the alignment is missing.

*Thank you, we appreciate that you pointed this out. We went through the manuscript again and aligned the text and altered the font sizes where needed.*

**Reviewer #2**

1. Which is prevalent cancer type among Dutch population based on the cancer incidence data reported between 2010 to 2022.

*Thank you for this question. The most common types of cancer in the country are cancer in the digestive systems, skin, breast lung and male genitalia. This is relatively constant across time. We discuss the most prevalent cancer types in the manuscript now. For your overview we added a graphical overview from the Netherlands Comprehensive Cancer Organisation below* *(*[*https://nkr-cijfers.iknl.nl/viewer/incidentie-per-jaar?language=en_GB&viewerId=2e7da421-e094-4f3f-aaab-a1369ae06cff*](https://nkr-cijfers.iknl.nl/viewer/incidentie-per-jaar?language=en_GB&viewerId=2e7da421-e094-4f3f-aaab-a1369ae06cff) *)*

Line 124

In the Netherlands, the most prevalent types of cancer are cancer in the digestive system and skin, followed by breast and lung cancer and finally, cancer in the male genital organs. General tendencies of cancer type incidence prevalence remains stable in the period of this work [37]. However, prevalence of a specific type of cancer does not predict news portrayals [38]. A bigger factor that determines if and how news media report on topics is how ‘newsworthy’ a topic is.

2. It is very difficult to make out the data from figure 3. Top left graph, what are those two trend lines in all the articles type data.

*Thank you for pointing this out. In line with the comment from reviewer 1, we exported our images again and converted them with the help of the tool the journal provided. The lines in all the plots in Figure 3 are trendlines In Figure 3.1 there are two trendlines as all articles and unique articles are compared. All other plots have one trendline as those are unique averages (as explained in the methods). We altered the legend for this image to explain this, as follows (Line 340):*

**Fig 3. Cancer screening coverage over time for all news per quarter (3.1) – differentiated between single articles and duplicates, and the current screening program consisting of breast (3.2), cervical (3.3), and colon (3.4) cancer, and early detection of lung (3.5) and prostate (3.6) cancer per month.** The absolute frequencies (bar graph) and rolling averages (line graphs) are shown.

3. This study provides data on Breast, colon, prostate, Lung and Cervical cancer. Does it mean Dutch media framed only these cancer types.

*Thank you for this comment. As we used a search string that related to cancer screening and populational screening and after that, we coded the data for inclusion, and we deliberately focused on the types of cancer screening you mention. We do this because breast, colon and cervical cancer screening are the current screenings that are offered as population screening. We focus on lung and prostate cancer (screening) because they are currently under debate within the government but also through the Dutch society, to be included into the populational screening programs. This means that in our sample we only have articles that focus on these types of cancer and screening. We do not have data on other types of cancer and how these are reported in news articles currently. We altered the explanation of this rationale to make it clearer in the methods section, as follows*

Line 246

Articles from Dutch newspaper articles were considered for inclusion when they reported on cancer screening programs currently in the Dutch populational screening program (namely, cervical, breast and colon cancer). We also included articles lung and prostate cancer screening as they were (and still are) under debate to be included in the populational screening program. Current methods included current screening practices (i.e., pap smear, mammogram, stool test) relevant for cervical, breast, and colon cancer. Debated methods included innovations such as curved paddles for mammograms.

4. In addition to the COVID pandemic situation, can authors implicate other situations leading to decline in breast cancer incidence.

*Thank you for this remark. As written above, the Netherlands Comprehensive Cancer Organization IKNL concluded that the decline in breast cancer incidence is predominantly due to the COVID-19 pandemic. The reason for this is that medical personnel was needed to focus on the pandemic. Therefore, the breast cancer screening program was paused, leading to less incidences of breast cancer.*

Line 570

Finally, we would like to point out that the COVID-19 pandemic had effects on our dataset. First, the incidence of registered breast cancer decreased during the pandemic. The reason for this is that the personnel that worked for the screening programs was allocated to help with the pandemic (e.g., to put less pressure on all people working in the Dutch healthcare system) [63].

5. To make the media to cover framing categories that might result in explicitly effective, what type of scientific data and information be disclosed from CSPs National Institute for Public Health and the Environment
   *Thank you for this comment. We now more clearly translate the findings that we have regarding the sources that frame explicit effectiveness in the discussion. In order to provide implications for future newspaper articles. These articles would, based on our data, best include health care providers, health organizations and people with personal experiences, as they are the sources that frame CSPs as explicitly effective the most. The National Institute for Public Health and the Environment mostly provides numbers for uptake and invitations on their website. We would recommend them to make the effectiveness of CSPs more tangible by also providing (implicating) numbers about the number of people that are cured because they took part in CSPs.*

*Line 523*

While one of the goals of the National Institute for Public Health and the Environment is to increase the participation in CSPs, we would advise the organization to, in collaboration with the newspaper publishers and journalists, involve health care providers and people with personal experiences in the articles. Moreover, the current websites of the institute mostly provide participation and invitation numbers, but the number of people that are cured (indirectly) because they took part in CSPs may be communicated more, so the effects can be made more tangible, and the benefits are clearer.

6. Figures: Figures are not presented with appropriate figure legends
   Figures are not provided in appropriate resolution, Images appear fuzzy as we zoom with texts are hazy.

*Thank you for this remark. We exported our images again and these are now in line with the journal standards.*

**Reviewer #3**

It is necessary for the author to refresh the current citations. The article's expectations are not met by this particular work. It is essential for the author to compile further data on the detrimental elements, especially those related to physiological issues, and to substantiate this with organizational evidence. Besides focusing on breast, colorectal, and cervical cancers, the editor could have explored additional forms of cancer.

*We appreciate this valuable comment. Our search strategy was intentionally designed to focus on the specific cancer screening types mentioned by the reviewer. We employed search terms related to cancer screening and population-based screening, followed by systematic coding for inclusion criteria that deliberately targeted breast, colorectal, and cervical cancer screening programs. This focus reflects the fact that these three screening modalities represent the currently established population-based screening programs. Additionally, we included lung and prostate cancer screening because these programs are presently under active consideration for implementation in national screening initiatives, both within governmental policy discussions and broader societal debate in the Netherlands. Consequently, our sample consists exclusively of articles addressing these five specific cancer screening modalities, which aligns with our study's objective to examine screening programs that are either currently implemented or under serious consideration for population-level implementation.*

*We added recent references to our manuscript:*

*Kaljouw, S., Jansen, E. E. L., Schevenhoven, V. J. C., & de Kok, I. M. C. M. (2025). Should the age range of the Dutch hrHPV-based cervical cancer screening program be broadened? A modelling study using cohort effects. International Journal of Cancer, 157(4), 627–633. <https://doi.org/10.1002/ijc.35435>*

*Galanos, L. J. K., Rijken, A., Elferink, M. A. G., Kok, N. F. M., van Erning, F. N., & De Hingh, I. H. J. T. (2025). Influence of a nationwide colorectal cancer screening program on the incidence of synchronous colorectal peritoneal metastases. International Journal of Cancer, 157(2), 232–238. <https://doi.org/10.1002/ijc.35356>*

*Luizaga, C. T. D. M., Câmara, A. B., Cury, L. C. P. B., Lopez, R. V. M., Contreras, C. A. H., Basu, P., Wünsch-Filho, V., & Carvalho, A. L. (2025). Barriers and attitudes toward cervical cancer screening among eligible women. Cancer Causes & Control. <https://doi.org/10.1007/s10552-025-02058-4>*

Line 246

Articles from Dutch newspaper articles were considered for inclusion when they reported on cancer screening programs currently in the Dutch populational screening program (namely, cervical, breast and colon cancer). We also included articles lung and prostate cancer screening as they were (and still are) under debate to be included in the populational screening program. Current methods included current screening practices (i.e., pap smear, mammogram, stool test) relevant for cervical, breast, and colon cancer. Debated methods included innovations such as curved paddles for mammograms.

**Reviewer #4**

1. Title - the title does not reflect the article content which includes media coverage of cancer as well as cancer screening; in addition it is important to reflect the type of media, which is limited to newspapers
   *Thank you for this remark. We altered our title to underline that our study focusses on newspaper coverage on both cancer screening and cancer. We put cancer screening first in the title to make sure we do not imply that we used data on every type of cancer in news articles, but that cancer screening and population screening is the primary focus of this study.*

“Unpleasant But Effective: Newspaper Coverage of Cancer Screening and Cancer in the Netherlands from 2010 to 2022”

2. The impact of the media coverage has not been sufficiently considered.

*Thank you for this remark, we would like to emphasize that this study did not aim to examine effects, rather we investigated the content of news media coverage. While we do agree that the impact of these news articles would be very interesting to investigate, this did not fall within the scope of the current study. We added your suggestion to our outlook for future research, as follows:*

Line 563:

Additionally, we would like to underline that within this focus on traditional news, we focused on the content of the news articles and do not have information about the impact that news articles have. While implications of media impact have been reported in other research [68,69], we build on that research and do not have new evidence regarding impact. Future research may be able to investigate the impact by for instance, looking at the frames that are used in articles on cancer screening and how individuals potentially adjust their attitudes because of this.

3. Who reads newspapers in the Netherlands? It is recognised in many countries that increasing numbers of people get their news from social media sources and newspaper reading is decreasing. What is the age profile of newspaper readers? The authors need to seriously consider this and reflect the situation in the Netherlands.

*Thank you for this comment. To better contextualize the study, we incorporate a short description of people reading newspapers in the Netherland. We now also reshaped our rationale based on the premise that parts of what is posted on social media is built on articles that are published in news media. Which shows the importance of analyzing news articles to map the broader societal discourse about CSPs. Moreover, we included the remark that indeed, age is a factor in news article consumption, but that this aligns with the fact that age is also a factor concerning cancer screening.*

Line 79:

Moreover, topics that are shared in news articles often overlap with topics that are discussed on social media platforms, which shows the relevance of investigating news articles in order to map the broader societal discourse about CSPs [22,23]. In the Netherlands, 29% of people over the age of 13 read newspapers [24], and there is a positive relation between peoples age and their newspaper reading[25]. This partially overlaps with the fact that people that are invited to participate in CSPs are usually of higher age [26,27].

4. Also the omission of social media in methods and discussion is unusual. Why not considerered in this analysis?

*Thank you for this comment. As discussed in the manuscript, we recognize that the role of social media in people’s media diet should not be underestimated. However, in this study we focused on newspaper articles. While other work investigating news consumption on social media regarding cancer screening would be very interesting and fitting in the current body of literature, this fell out of the scope for the current study. In line with the answer above we now reflect on the overlap of news media consumption and social media discourse in the Theory, moreover we added this in our discussion where we discuss this point to make this clearer. Finally, while it is not in the paper, we would like to point out that we indeed, are writing up two studies in this project where we look at online discourse on Twitter and on one of the country’s largest forums and investigate how information is discussed there regarding the CSPs with various mixed methods.*

Line 553

With this work, we aimed to describe and scrutinize how written news media reports in the Netherlands talk about cancer and cancer screening in the country. While news media still play an important in setting the public agenda, research shows that this agenda is often overlapping with social media discussions, also in the case of cancer-related health communication [22,23]. Moreover, it is important to recognize that parts of the public do use social media platforms as their prime source of news media, either directly or by proxy (i.e., following news production companies or outlets on Facebook or Twitter). Thus, next to news media reports, future research could aim to draw stronger comparisons between traditional news and social media occurrences of cancer and cancer screening and information gathering or even participation in cancer screening.

5. The article is long and some editing would be useful. Sentence structure editing is needed in places.

*Thank you for pointing this out. For the revised version we edited the sentence structure in various places.*

6. Figures do not appear to be correctly numbered or titled at the end of the document

*Thank you for this comment, we indeed had some issues with the figures, they are now resolved and therefore should be correctly numbered and titled in the manuscript*.

**Reviewer #5**

1. Search procedure:
   As mentioned, non-Dutch newspapers were excluded. However, the manuscript does not clarify whether scientific English-language articles from Dutch newspapers were considered. If no such English-language content exists, please state this explicitly.
   Additionally, if there was uncertainty about this, it may have been helpful to include English keywords (e.g., cancer, colorectal cancer, colposcopy) in the search strings as well.

*Thank you for pointing this out. We did not include articles written in English. Moreover, no English written articles were found in the dataset. We now clarify this better in the methods (search procedure)*

Line 202
We excluded English-written news articles.

2. Discussion:
   Lines 456 to 458: The comparison was insightful, However, the information would be more accessible if presented visually — e.g., as a diagram or chart.

*Thank you for this remark, the volume/ratios of articles reporting on cancer and cancer screening can be found in figure 2.*

3. Lines 470 to 472: Please clarify the contrast mentioned in the sentence: “In addition, our results were also in contrast with other work showing that cancer screening search trends decreased during the COVID-19 pandemic.” Based on both your data and previous studies, it seems that cancer-related news coverage declined during the COVID-19 pandemic, regardless of the country. If this is the case, there may not be a real contrast. Please elaborate on how your findings differ.
   *Thank you for this comment. We have explained our rationale better in the manuscript. Our data show no large increase or decrease if we look at reports on cancer and cancer screening (Fig. 3.1). This is what we mean with this part of the discussion section. We explained this as follows:*

Line 489

In addition, our results were also in contrast with other work showing that cancer screening search trends decreased during the COVID-19 pandemic [60]. Our data shows that the volume of news reports around the outbreak of the pandemic remained stable. Conversely, news reporting on breast cancer screening increased because the screening program was paused because of the pandemic. A broader reason for this contrasting finding might be that news consumption increased during the pandemic, but only for television, Internet, and social media [58].

4. Acknowledgements:
   This section would benefit from clearer justification and improved wording to align stylistically with the rest of the manuscript.

*Thank you for this remark, we now altered our acknowledgements based on comments of the reviewer, they should now be aligned with the rules of the journal.*

Line 587
We thank Lisa Salm and Noa ter Braak for their assistance with the data coding.

Declaration of interest statement: the authors declare no potential conflicts of interest with respect to the research, authorship, and/or publication of this article.

Data availability statement: raw files, study protocol, analysis plan, and codebook can be found on OSF: <https://osf.io/z7y6u/>

5. Table 4:
   The categories Preventive Measures and Sources appear inconsistent in terms of total values and percentages.
   Additionally, Alternative screening (e.g., full-body scan), mentioned in Line 246, is missing from the table. Please review and adjust the totals and percentages accordingly.

*Thank you for this remark, we have ensured all subvalues divided by the N (675) give the correct percentages. Looking at the total scores, it is possible that some sources or measures are not mentioned in every article, and oppositely, that others are mentioned double. We now clarify this in the manuscript in the subnote of Table 4, as follows:*

Line 322
*NB: It is possible that some subtotals are lower or higher than the total of the category because a frame could occur multiple times in an article (e.g., cancer consequence the subtotal is higher than the total frequencies and preventive measures is lower).*

6. Table 5:
   Cancer consequences: Neutral: The total amount should be corrected to 300 (53.5)
   *Thank you for pointing this out, we have altered this.*
7. Table 6:
   Cancer risk: Please double-check and reconsider the calculation.
   *Thank you for this remark, we have altered this subgroup, it is now correct in the manuscript.*
8. Table 7:
   Screening criteria: Cons: The statement “Those with a positive test result do not have any guarantees of not having colon cancer. The current test is expected to miss one in three cases.” may be misleading. If the point is about false negatives or limited sensitivity, the phrase should reference negative test results instead.
   *Thank you for pointing this out, we see that indeed, something happened in translation and that this should have been worded as a negative test result. We altered this in the manuscript.*

**Reviewer #6**

Introduction

1. Lines 87-96: I wonder if these differences in findings could be due to cultural differences. Do the authors have any thoughts on this?

*Thank you for this helpful remark. We now contextualize these findings from the literature better by explaining that cultural differences may moderate them. We added a reference showing that indeed, white Europeans (British, who were at the time of that study still Europeans)* *are more persuaded by gain-framed health messages, while East-Asian participants had a stronger prevention focus and were more strongly persuaded by a loss-framed message.*

Line 103

It should be noted that these contrasting results could be moderated by cultural differences, as other research shows that white Europeans are more persuaded by gain-framed health messages, while East-Asian participants have a stronger prevention focus and are more strongly persuaded by a loss-framed message [31].

2. Lines 105-108: Typos/awkward wording, suggest rewording as: “Taking the above into account, the first aim of this work is to describe cancer related news, based on its volume and characteristics. Moreover, we aim to describe the frames used in cancer related news based on the pros and cons of screening that are presented, and which arguments are used for pros and cons. Combined, this is formulated in our first research question:”

*Thank you for this remark. Based on your comment and one from another reviewer we changed the wording in the passage leading up to RQ1.*

Line 115

Taking the above into account, the first aim of this work is to describe cancer related news, based on its volume and characteristics. Moreover, we aim to describe the frames used if pros and cons of screening are presented, and subsequently, which arguments are used for these presentations. Combined, this is formulated in our first research question:

3. Sentence beginning on line 116: My understanding is that breast cancer is the most common type of cancer in women, so is it really overrepresented? I think this point would benefit from a bit more detail, e.g., “overrepresented compared to its relative frequency” if that is the case.

*Thank you for this comment. This is partially the case. While it used to be the second most diagnosed type of cancer (until 2010), breast cancer is among the top 5 most occurring types of cancer (e.g., in 2024 it was 4^th^) but the incidence numbers differ a lot. In the Netherlands the most occurring types of cancer are skin cancer (25,281) and cancer in the digestive system (22,602) while breast cancer was diagnosed 15,585 times, just a bit less than prostate and testicular (bundled as ‘male reproductive’) cancer16,309. To make our statement clearer, we use your suggestion and make the statement in the manuscript more explicit (for more information please see* [*https://nkr-cijfers.iknl.nl/viewer/incidentie-per-jaar?language=nl_NL&viewerId=b6d90fa4-c3f0-469b-85d5-4d7281ccabb6*](https://nkr-cijfers.iknl.nl/viewer/incidentie-per-jaar?language=nl_NL&viewerId=b6d90fa4-c3f0-469b-85d5-4d7281ccabb6) *for a graphical overview of incidences).*

Line 124

In the Netherlands, the most prevalent types of cancer are cancer in the digestive system and skin, followed by breast and lung cancer and finally, cancer in the male genital organs. General tendencies of cancer type incidence prevalence remains stable in the period of this work [37]. However, prevalence of a specific type of cancer does not predict news portrayals [38]. A bigger factor that determines if and how news media report on topics is how ‘newsworthy’ a topic is.

Line 132

Research shows that news coverage over time differs as well, potentially contributing to overrepresentations, for instance regarding breast cancer. While breast cancer is one of the most common types of cancer, research shows that it is overrepresented compared to its relative frequency and mortality rates [37,38]. Work on illness representations demonstrates that people develop their own (mis-)representations of the illness, which may partially be based on overrepresentations in news media [40]. This shows that indeed, the public perceptions of cancer do not always represent the reality of the disease.

4. Last paragraph on page 6, wording suggestions to make slightly more formal, starting at line 123: “However, to the best of our knowledge, no work that investigates news media reporting on cancer in the Dutch media landscape has yet been published. Therefore, the second aim of this work is to describe the coverage of cancer screening in written news media over time from 2010 until 2022. The third aim is to investigate differences in reporting between the three types of cancer screening. These aims lead to the second and thirs research questions:”

*Thank you, this was very a very helpful suggestion to indeed change the tone of the summary leading to RQ2 and RQ3. We changed the wording in our manuscript.*

Line 140

However, to the best of our knowledge, no work that investigates news media reporting on cancer in the Dutch media landscape has yet been published. Therefore, the second aim of this work is to describe the coverage of cancer screening in written news media from 2010 till 2022. The third aim is to investigate differences in reporting between the three different types of cancer screening. These aims lead to the second and third research questions:

5. Line 131: RQ2 should start with either “to what extent” or “how,” or there should be an “and” that is missing.

*Thank you, based on another review we indeed saw that the wording was incorrect. We changed this accordingly.*

Line 147

RQ2: How does the coverage in newspaper articles change over time?^[1]^

6. Line 151: Can you use a more specific word than “things”?

*Thank you, we altered the sentence to better show our arguments.*

Line 167

However, these authors argue, that the newsworthiness of new methods could also backfire, as coverage of new screening technologies could undermine public confidence in the current and established CSPs, that are proven to be effective [25].

7. First full paragraph on page 8: Do you need “and pros and cons”? I thought this was how frames were being characterized.

*Thank you for this comment, we altered the wording accordingly. Showing our rational clearer, while focusing more on what we did, instead of just pro’s and cons.*

Line 170

The above shows that it is not only important to unravel which frames regarding cancer screening are discussed in news media reports, but also to investigate how these frames are contextualized to better understand how cancer screening is portrayed in news media reports. We will investigate information about CSPs in news articles based on which sources are used in the article (e.g., scientists or people with experiential knowledge) and how these sources discuss the CSPs. Further, we will assess whether frames are positive or negative about current (vs. innovative) measures via the presented pros and cons in the articles.

8. I think more detail is needed in either the description or specific wording of RQ4. Can you tell us what you mean by “how information is contextualized”? Does this mean who is being quoted in the articles? What articles appear next to it? Needs a little more explanation/clarity.

*Thank you for this remark, we would like to reply to both comments. We coded using two constructs. First, we coded if different sources such as healthcare professionals, scientists or individuals with experiential knowledge, in news articles framed cancer screening differently. To do so, we investigated whether they, for instance, framed screening as explicitly or implicitly effective. Further, we assessed whether arguments were positive or negative about the current (vs. innovative) measures. We then grouped those arguments, resulting in categories discussing: the screening criteria, screening organization, the physical impact of screening and the psychological context. We altered the paragraph leading to RQ4, as well as RQ4 itself, to better reflect the analyses we conducted and how we discuss the framing and contextualization of the CSPs in the news articles.*

Line 170

The above shows that it is not only important to unravel which frames regarding cancer screening are discussed in news media reports, but also to investigate how these frames are contextualized to better understand how cancer screening is portrayed in news media reports. We will investigate information about CSPs in news articles based on which sources are used in the article (e.g., scientists or people with experiential knowledge) and how these sources discuss the CSPs. Further, we will assess whether frames are positive or negative about current (vs. innovative) measures via the presented pros and cons in the articles. Therefore, the fourth and final aim of this work is to investigate how information about cancer screening is contextualized in written news articles, leading to the fourth research question:

RQ4: How is cancer screening portrayed in news reports with respect to the sources cited and the frames employed?

Method

9. - Search procedure, line 173: Suggest adding the month in 2010 when search started (January?) since you include the month in 2022.
   - Line 235: Should “news” be “news reports”?
   - I appreciate the thoroughness of this section.

*Thank you for the suggestions and the appreciation, we have included your suggestions in the manuscript.*

Results

10. - Line 318: Suggest rewording to “Though this proportion may seem high, only a few articles explicitly reject screening…”
    - Line 402: Should “neutral” be “as neutral” or “neutrally”?
    - Line 428: Suggest rephrasing, awkward to start the sentence with “Meaning that…”

*Thank you for these remarks. We changed the wording and replaced neutral with neutrally. Finally, we altered “Meaning that…” with “This shows that…”. Alterations can be found in the manuscript.*

Discussion

11. 20. Line 457: I don’t think you need the parentheses around “much.”
    21. Line 460-463: Suggest splitting into two sentences.
    22. Line 462: I think the word “can” should be “may.”
    23. Line 493: Typo, “frames” should be “framed.”
    24. Line 507: The suggestion to include celebrities in CSPs seems slightly out of place. I suggest either removing this or digging into the idea a bit more.
    25. Lines 509-11: Similarly, I would either remove the point about informed consent or go into more detail on it.
    26. Line 516: Typo, “than” should be “then.”

*Thank you for these remarks. We changed the points that related to copy-editing, and we removed the point about celebrities and the point about informed consent, as the paper is/was relatively long already. Alterations can be found in the manuscript.*

Figures
27. Figure 2: Would it be possible to have the place the N for cancer consistently to the right of the shaded bar? Having two different places for this N is confusing.

*Thank you for this remark, we have altered the image and now, the N’s for cancer are on the right side of the bars.*

1. This research question differs from the one we preregistered, as we do not investigate the extent, we focus on that in RQ3.
